# Clinician perspectives on virtual reality use in physical therapy practice in the United States

**DOI:** 10.1371/journal.pone.0320215

**Published:** 2025-04-02

**Authors:** Danielle T. Felsberg, Jared T. McGuirt, Scott E. Ross, Louisa D. Raisbeck, Charlend K. Howard, Christopher K. Rhea

**Affiliations:** 1 Department of Kinesiology, University of North Carolina at Greensboro, Greensboro, North Carolina, United States of America; 2 Department of Physical Therapy, Wingate University, Wingate, North Carolina, United States of America; 3 Department of Nutrition, University of North Carolina at Greensboro, Greensboro, North Carolina, United States of America; 4 Ellmer College of Health Sciences, Old Dominion University, Norfolk, Virginia, United States of America.; La Trobe University, AUSTRALIA

## Abstract

The primary goal of physical rehabilitation is to assess movement impairments and restore function to improve overall quality of life. Virtual reality (VR) may provide the optimal environment to promote these goals due to its motivating and modifiable nature which can be difficult to accomplish through traditional real-world therapeutic methods. Current research of VR for rehabilitation has demonstrated that VR interventions can produce clinically meaningful change in motor outcomes. Despite this, adoption and usage of VR by physical therapy professionals is unclear due to the limited research in this area. Thus, the purpose of this study was to identify the current usage and perspectives of VR in physical rehabilitation among physical therapy professionals. Physical Therapists (PTs) and Physical Therapist Assistants (PTAs) in the United States were recruited to participate in this survey-based study. A total of N = 658 participants completed the survey, which consisted of demographic information followed by the Assessing Determinants Of Prospective Take-up of Virtual Reality (ADOPT-VR2) survey that assesses 12 constructs (e.g., Attitudes, Perceived Usefulness, Facilitating Conditions and Barriers) related to the use of VR in clinical settings. Most respondents reported not using VR in clinical practice (n = 611; 92.9%). For all respondents, the constructs of Attitudes, Perceived Ease of Use, Compatibility, Client Influence, and Self-Efficacy were found to statistically contribute to the prediction of Behavioral Intention to use VR (p < .05). However, for those clinicians using VR in their clinical practice, Superior Influence and Perceived Behavioral Control were significant positive predictors for intention to use VR. Future investigation should aim to test strategies to target these factors significantly influencing VR use to further address the gap between evidence and clinical practice in the use of VR for physical rehabilitation interventions.

## Introduction

The primary goal of physical rehabilitation is to assess movement impairments and restore function with the goal of improving quality of life following aging, injury, or disease. A growing body of literature demonstrates that virtual reality (VR) has utility in healthcare settings in general [1], and more specifically, can enhance the assessment and rehabilitation of movement [2–7]. VR can be defined as an artificial, computer-generated environment which contains sensory information that users can interact with, visualize, and (or) manipulate to allow natural behaviors to emerge as though the environment were real [8–10]. VR affords the ability to create safe, multisensory, modifiable, and/or interactive interventions, and these unique features of VR may provide the optimal environment to promote the learning, retention, and transfer of motor skills in a way that is difficult to accomplish through traditional therapeutic methods. For example, it has been shown that VR can be used to modify fractal gait patterns (i.e., low-level biological variability associated with functional mobility) over a period of 10 minutes of training [11]. An intervention of that nature with visual stimuli would be nearly impossible to create in the real world. Moreover, VR can be used to produce interventions that incorporate elements of observational learning, practice, feedback, and motivation in ways that align with the mission of physical rehabilitation. Current literature demonstrates that VR interventions can improve motor outcomes, and these improvements can even be superior to those seen following standard physical therapy intervention [4–6]. Some examples of the VR devices used are the HTC Vive, Meta Quest, Xbox Kinect, and Wii Fit. Additionally, given the technology and attributes of VR hardware and software, these key motor learning features typically employed in rehabilitation interventions can be executed in a precise, timely, and customizable way that is difficult to achieve in the physical environment, which even further supports the potential incorporation of VR in clinical settings.

A variety of VR technologies exist on the market at a range of price points, available features, and levels of immersion to fit the specific needs of various clinical settings. As technology has developed, and VR has become more widely available, clinicians and researchers alike have identified VR as a potentially useful tool in providing rehabilitation interventions to improve motor function. Current research in this area of VR for rehabilitation has largely demonstrated that VR interventions can produce clinically meaningful changes in motor outcomes [2,3,5–7]. Although the research in this area has increasingly been moving out of controlled-laboratory settings and into clinical settings, adoption and use of VR by clinicians has been moderate at best [12–16].

Although it has been shown that VR interventions can enhance motor outcomes, there appears to be a gap between evidence and practice. The current evidence supports the use of VR for rehabilitation, but that does not seem to translate to actual VR use by practicing clinicians [4,14,15]. Grounding these observations in theoretical behavioral models can help guide research questions and data interpretation. Several theories and models exist to identify the determinants of technology adoption. These include the Technology Acceptance Model (TAM) [17], Diffusion of Innovation Theory [18], and Decomposed Theory of Planned Behavior (DTPB) [19]. Together, these models help to define how various factors like perceived usefulness, perceived ease of use, behavioral control, and social influences impact an individual’s attitude toward technology. These models then aid in the understanding of how an individual’s attitudes toward technology, in this case VR, influence their acceptance and ultimate usage of technology.

Previous research in the area of VR acceptance by clinicians led to the creation of the Assessing Determinants of Prospective Take-up of Virtual Reality survey (ADOPT-VR2). Two studies have surveyed groups of both physical therapists (PTs) and occupational therapists (OTs) in Canada and the United States (US) [14,15] using the ADOPT-VR2 and showed a moderate amount of therapists have had experience with using VR in their clinical practice (Canada = 46%; US = 64%), but the number of clinicians using VR in their current practice is considerably lower (Canada = 12%; US = 31%) [15]. Data from these studies showed the ADOPT-VR2 constructs of attitudes toward VR (i.e., perceived ease of use, perceived usefulness, compatibility of VR to the therapist’s clinical practice, social norms, peer influence, client influence, therapist self-efficacy, and facilitating conditions) were all predictive of intention to use VR [14,15]. However, the VR landscape with respect to cost, accessibility, and public awareness has changed since the time these two studies were conducted in 2014–2015 [14] and 2017–2018 [15]. Notably, VR technology became more readily available by the consumer in the early 2010s with the debut of the original Oculus head-mounted displace (HMD). Prior to the Oculus, the most popular way for consumers to interact with virtual games and environments was through the Nintendo Wii (2006) and Xbox Kinect (2010). VR technology has accelerated at a rapid pace over the past 15 years and perceptions of VR are likely to be influenced by the time window in which they were assessed. Another limitation is that these surveys included both OTs and PTs which, although similar professions, often have different primary goals and interventions. For example, OTs commonly focus on fine motor skills, upper extremity tasks, and performing activities of daily living such as dressing. However, PTs commonly focus on gross motor skills, motor performance, and functional tasks such as ambulation. Thus, VR interventions for each focus maybe different. Additionally, both professions have assistant-level disciplines providing treatment to patients. For PTs, Physical Therapist Assistants (PTAs) play a critical role in executing treatment plans to meet the goals set by the treating therapist which could include the use of VR. Understanding PTA perspectives would provide valuable insight into adoption of VR and barriers to use.

At present, it is unknown the extent to which VR is being used in clinical settings for physical rehabilitation specifically by PTs and PTAs. However, the literature suggests that VR usage in clinical practice is not commensurate with the evidence supporting the utility of VR interventions to improve physical function. To assess the current clinical use of VR by PTs and PTAs, theory driven survey-based research regarding technology acceptance of licensed clinicians could help explain why adoption of VR may be low in clinical settings despite the evidence in support of its use. Thus, the purpose of this study was to identify the acceptance, adoption, and perceived barriers of use of VR in rehabilitation by physical therapy professionals.

## Methods

The Institutional Review Board (IRB) at the University of North Carolina at Greensboro (UNCG) approved this study (IRB-FY21–263). All participants provided written informed consent.

PTs and PTAs practicing in the United States were recruited to participate in this cross-sectional survey-based study. Recruitment was a convenience sample consisting of e-mail contact through both state licensing boards and specialty sections of the American Physical Therapy Association (APTA). Participants were also recruited via convenience and snowball sampling using social media and other digital outlets. There were no incentives given for survey completion. All participants completed an online, IRB-approved informed consent process, including the estimated time to complete the survey and information regarding the anonymity of their responses. The survey was first disseminated on 07/01/2021, monitored for responses, and recirculated as needed through 12/31/2021. No names, e-mail addresses, or electronic identifiers were collected, thus all survey responses were anonymous and identified only by responses number.

The survey sent to clinicians consisted of demographic information followed by the ADOPT-VR2 survey [14,15]. The demographic portion of the survey included questions related to age, professional status, education level, clinical experience, and experience with using VR in clinical practice. Questions regarding patient population, patient age, and purpose of VR interventions allowed respondents to select multiple responses that applied to them. The ADOPT-VR2 consists of 53 questions that address 12 constructs: Attitudes (3 questions): Perceived Usefulness (3 questions), Perceived Ease of Use (3 questions), Compatibility (2 questions), Social Norms (2 questions), Client Influence (1 question), Peer Influence (2 questions), Superior Influence (2 questions), Perceived Behavioral Control (4 questions), Self-Efficacy (13 questions), and Facilitating Conditions and Barriers (15 questions). The questions for each construct were asked on a 9-point Likert scale where 1 was “strongly disagree” and 9 was “strongly agree”. A higher score indicates stronger alignment with using VR in clinical practice. Scores for each construct were calculated as the average value across the responses within each construct. The ADOPT-VR survey has been found to have strong face and content validity, and good internal consistency (Cronbach’s alpha =  0.876) [12].

We were also interested in the extent to which participants reported their intention to use VR after watching a short video on the use of VR in clinical practice at the end of the survey. This was motivated by theoretical framework of Behavior Change Techniques (BCT) that can be described as the smallest active ingredient(s) that can change behavior in an intervention [20]. Accordingly, following the initial survey, participants were presented with an informational video providing examples of VR use in physical rehabilitation. The video was a short recording (3 minutes, 55 seconds) of a PowerPoint slideshow that defined VR and identified different factors that influence the VR experience, along with examples of VR. A link to the video can be found in the Supplementary Material (S1 Link). Although our video would not be considered an “intervention”, it did provide information that participants could use to make a decision about their future intentions. After viewing the video, participants were asked two ‘yes/no’ questions: (1) “Now knowing more about VR for physical therapy, do you plan to use VR in the future?” and (2) “Now knowing more about VR for physical therapy, do you intend to seek more clinical education regarding VR?”.

The survey responses were collected online using REDCap. Data gathered from the demographic portion of the survey were analyzed using descriptive statistical methods as appropriate. A multiple regression analysis with simultaneous entry was conducted to explore the extent to which the 11 independent variables (i.e., the behavioral constructs of Attitudes, Perceived Usefulness, Perceived Ease of Use, Compatibility, Social Norms, Client Influence, Peer Influence, Superior Influence, Perceived Behavioral Control, Self-Efficacy, and Facilitating Conditions and Barriers) predicted our dependent variable of Behavioral Intention to use VR in clinical practice. Behavior Intention refers to the extent to which a person plans to adopt a particular action. Using the aforementioned 11 behavior constructs to predict Behavior Intention aligns with previous research that used the ADOPT-VR2 survey to explore motivation to (or not to) incorporate VR in clinical practice [14,15,21,22]. A secondary analysis, binary logistic regression, was conducted to ascertain the associations of 11 behavioral constructs with the likelihood of clinicians using VR in their practice (yes/no). An alpha of.05 was set for all statistical analyses. No corrections were made to the p-value since the multiple regression procedure controls for multiple variables at the same time [23]. Statistical analyses were performed using the statistical program SPSS version 29 (IBM, Chicago, IL, USA).

## Results

A total of 1,007 people initiated the survey. Of these, 658 clinicians (PTs =  555; PTAs =  103) completed the full survey. Thus, the completion rate for the survey was 65.3%. Only the 658 fully completed survey responses were used for statistical analysis in this study (S2 Table). Demographic information for all participants is shown in Table 1.

**Table 1:** Participant demographic information.

Participant Demographics		Total Sample (n = 658) n(%)	PTs (n = 555) n(%)	PTAs (n = 103) n(%)
**Age**	**20–29**	65 (9.8)	58 (10.4)	7 (6.8)
	**30–39**	152 (23.1)	128 (22.9)	24 (23.3)
	**40–49**	149 (22.6)	127 (22.9)	22 (21.4)
	**50–59**	182 (27.7)	151 (27.2)	31 (30.1)
	**60–69**	103 (15.7)	84 (15.1)	19 (18.4)
	**>70**	7 (1.1)	7 (1.1)	**–**
**Geographic Region**	**Northeast**	52 (7.9)	46 (8.3)	6 (5.8)
	**Southeast**	304 (46.2)	269 (48.5)	35 (34.0)
	**Midwest**	159 (24.2)	119 (21.4)	40 (38.8)
	**Southwest**	123 (18.7)	104 (18.7)	19 (18.4)
	**West**	20 (3.0)	17 (3.1)	3 (2.9)
**Education Level** **(Highest completed)**	**Associate’s**	51 (7.6)	–	51 (49.5)
	**Bachelor’s**	149 (22.6)	107 (19.3)	42 (40.8)
	**Master’s**	135 (20.5)	127 (22.9)	8 (7.8)
	**Clinical Doctorate**	297 (45.1)	296 (53.3)	1 (1.0)
	**Terminal Doctorate**	26 (4.0)	25 (4.5)	1 (1.0)
**Recreational Use of VR**	**Yes**	48 (7.6)	35 (6.3)	15 (14.6)
	**No**	610 (92.4)	520 (93.7)	88 (85.4)
**Explored VR at Work**	**Yes**	93 (14.1)	77 (13.9)	16 (15.5)
	**No**	565 (85.7).	478 (86.1)	87 (84.5)
**Clinical Use of VR**	**Yes**	47 (7.1)	38 (6.8)	9 (8.7)
	**No**	611 (92.9)	517 (93.2)	94 (91.3)
**Employer Affiliation with Research Institution, University or Teaching Hospital**	**Yes**	190 (28.9)	169 (30.5)	21 (20.4)
	**No**	468 (71.1)	386 (69.6)	82 (79.6)

**Northeast:** Maine (ME), New Hampshire (NH), Vermont (VT), Massachusetts (MA), Connecticut (CT), Rhode Island (RI), New York (NY), Pennsylvania (PA), New Jersey (NJ) **Southeast**: Maryland (MD), Delaware (DE), Virginia (VA), West Virginia (WV), Kentucky (KY), Tennessee (TN), North Carolina (NC), South Carolina (SC), Georgia (GA), Alabama (AL), Mississippi (MS), Florida (FL), Arkansas (AR), Louisiana (LA) **Midwest:** North Dakota (ND), South Dakota (SD), Nebraska (NE), Kansas (KS), Minnesota (MN), Iowa (IA), Missouri (MO), Wisconsin (WI), Illinois (IL), Michigan (MI), Indiana (IN), Ohio (OH) **Southwest**: Arizona (AZ), New Mexico (NM), Oklahoma (OK), Texas (TX) **West:** Hawaii (HI), Alaska (AK), Washington (WA), Oregon (OR), California (CA), Nevada (NV), Idaho (ID), Montana (MT), Wyoming (WY), Utah (UT), Colorado (CO).

**Fig 1:**
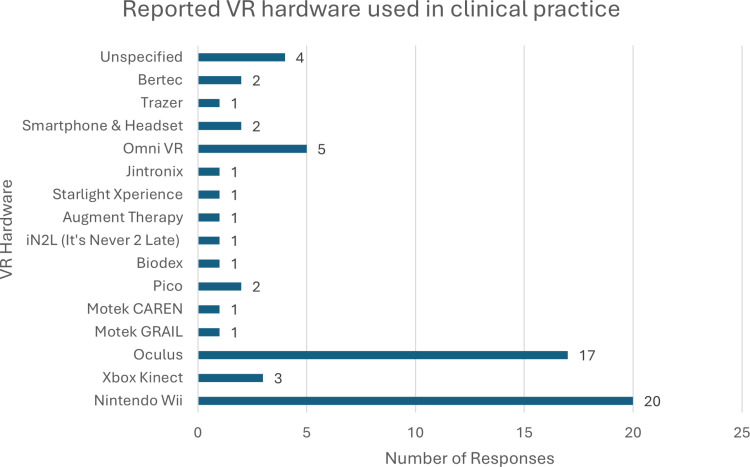
VR hardware used in clinical practice. Types and frequences of the VR equipment reported to be integrated in clinical practice.

Information regarding current use of VR in clinical practice can be found in Table 2. Geographically, the majority of respondents reported practicing in the southeast region (n = 304), and the lowest number of respondents were from the western region (n = 20). Of note, recruitment was done via convenience and snowball sampling using e-mail contact and other digital outlets, like social media. The states of Arizona, Louisiana, Maine, North Carolina, Ohio, and Wyoming freely provided contact information for licensed clinicians in their states, allowing for more targeted recruitment from these areas. Forty-seven respondents (7.1%) reported using VR in their clinical practice. Most participants reported using VR in outpatient settings (n = 26) followed by sub-acute rehabilitation (n = 11) and acute rehabilitation (n = 3) settings. The four most common patient populations respondents used VR interventions with are Neurologic (n = 33), Orthopedic (n = 31), Sports (n = 14), and Cardiopulmonary (n = 12). Participants reported using VR the most with adult populations. Of the clinicians using VR in their practice, most used it for purposes related to neuromuscular reeducation (n = 32), balance (n = 32), as a motivator (n = 22), or for gait training (n = 18). The types of VR hardware used in clinical practice can be found in Fig 1. To perform these interventions, consumer products like Nintendo Wii (n = 20) and Oculus (n = 17) were most commonly used.

**Table 2:** Virtual reality use in clinical practice.

		Total Sample (n = 47)	PTs (n = 38)	PTAs (n = 9)
**Setting**	**Acute Care**	2	2	0
	**Acute Rehab**	3	2	1
	**Sub-acute Rehab**	11	7	4
	**Outpatient**	26	24	2
	**Home Health**	2	2	0
	**Long-term Care**	2	0	2
	**Academic**	1	1	0
**Patient Population**	**Orthopedic**	31	25	6
	**Neurologic**	33	26	7
	**Cardiopulmonary**	12	8	4
	**Sports**	14	12	2
	**Pelvic Health**	2	2	0
	**Pediatrics**	2	1	1
	**Geriatrics**	2	2	0
	**Chronic Pain**	3	2	1
	**Vestibular**	2	2	0
	**Not Specified**	3	2	1
**Patient Age**	**<2 years**	0	0	0
	**2–5 years**	1	1	0
	**6–12 years**	14	12	2
	**13–18 years**	23	21	2
	**19–44 years**	30	26	4
	**45–64 years**	35	29	6
	**65–79 years**	34	28	6
	**>80**	29	25	4
**Purpose**	**Balance**	32	23	9
	**Strength**	14	10	4
	**Range of Motion**	14	8	6
	**Gait**	18	12	6
	**Neuromuscular Re-ed**	32	23	9
	**Motivation/Reward**	22	15	7
	**Other:** Education	5	5	0
	External Focus	1	1	0
	Engagement	1	0	1
	Endurance	2	1	1
	Vestibular Stim.	1	1	0
	Relaxation	1	1	0
	Coordination	2	1	0
	Cognition	1	0	1
	Implicit Learning	1	1	0
	Mindfulness	3	3	0
	Breathing Exercises	3	3	0

**Fig 2:**
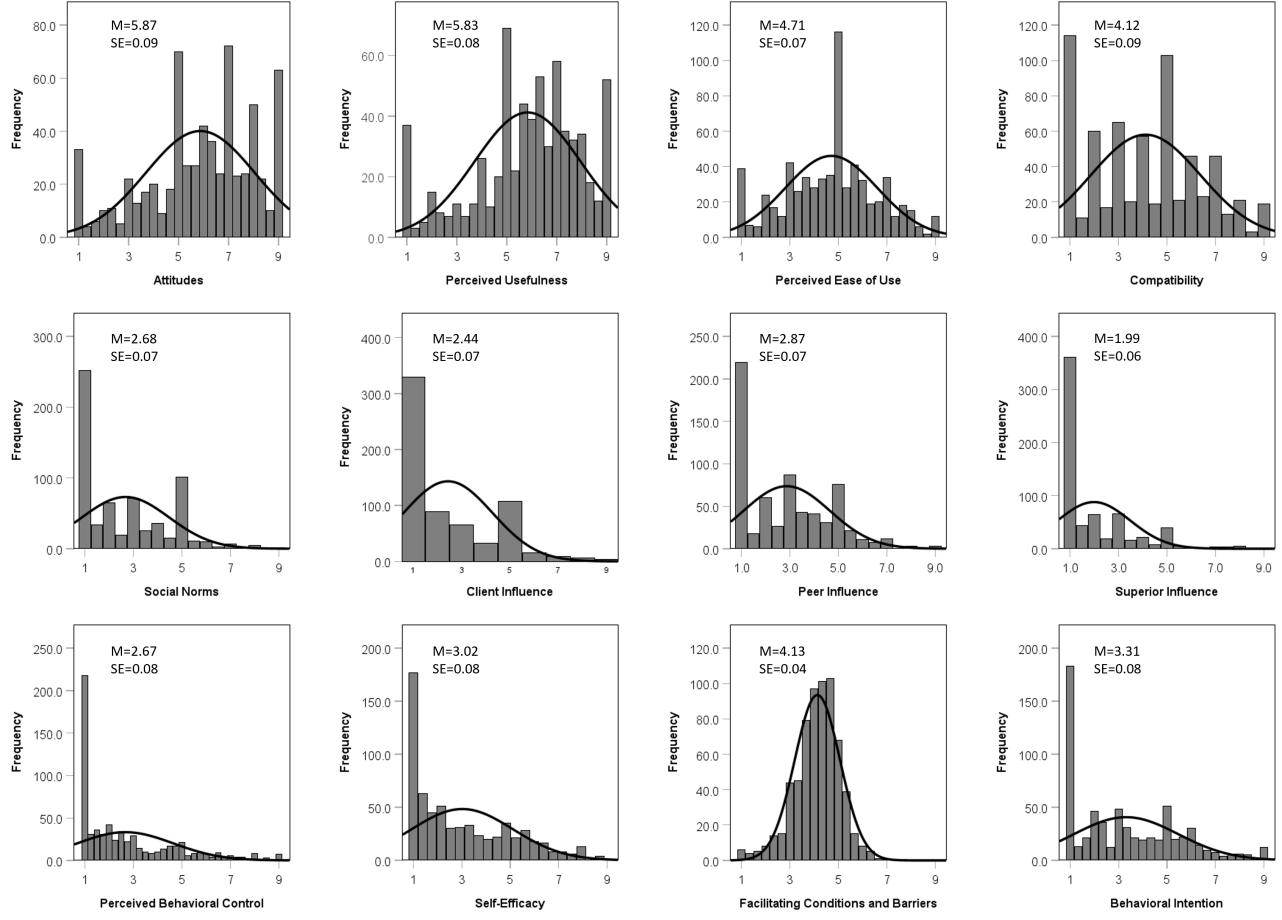
Histogram of responses for each of the constructs included in the ADOPT-VR2 survey. The questions for each construct were asked on a 9-point Likert scale, where 1 was “strongly disagree” and 9 was “strongly agree”. Scores for each construct were calculated as the average value across the responses within each construct. SE =  standard error.

The mean responses for each construct of the ADOPT-VR2 can be found in Fig 2. To determine the predictive value of the ADOPT-VR2 constructs on behavioral intention to use VR, a multiple regression was performed. No assumptions were violated. The model was statistically significant, F(11,646) =  91.049, p < .001, with an R^2^ of.608. Of the 11 behavioral constructs included in the model as independent variables, five were found to statistically contribute to the prediction of Behavioral Intention to use VR (p < .05). This included Attitudes, Perceived Ease of Use, Compatibility, Client Influence, and Self-Efficacy (Table 3). Partial correlations are also presented in Table 3 to indicate the direction and magnitude of the relationships for each construct.

**Table 3:** Results of the multiple regression analysis with simultaneous entry.

	Unstandardized		Standardized Beta	t	p	95% Confidence Interval		Partial Correlation
Variable	Beta	SE				Lower Bound	Upper Bound	
Attitudes	0.145	0.056	0.147	2.567	0.010^*^	0.034	0.256	0.101
Perceived Usefulness	0.101	0.055	0.100	1.844	0.066	−0.007	0.209	0.072
Perceived Ease of Use	−0.088	0.036	−0.078	−2.440	0.015^*^	−0.159	−0.017	−0.096
Compatibility	0.310	0.040	0.326	7.811	<.001^*^	0.232	0.388	0.294
Social Norms	0.047	0.058	0.039	0.816	0.415	−0.066	0.160	0.032
Client Influence	0.109	0.055	0.103	2.204	0.028^*^	0.013	0.229	0.086
Peer Influence	0.077	0.050	0.039	0.928	0.354	−0.052	0.145	0.036
Superior Influence	0.162	0.057	0.076	1.906	0.057	−0.003	0.222	0.075
Perceived Behavioral Control	0.020	0.042	0.071	1.820	0.069	−0.006	0.160	0.071
Self-Efficacy	0.162	0.041	0.157	3.984	<.001^*^	0.082	0.241	0.115
Facilitating Conditions & Barriers	0.020	0.061	0.009	0.328	0.743	−0.100	0.140	0.011

Note.

* p < .05.

For the binary logistic regression analysis, Superior Influence (p = .029), and Perceived Behavior Control (p < .001) emerged as statistically significant variables. These results suggest that these variables play a significant role in influencing clinicians’ utilization of VR in their practice (as shown in Table 4).

**Table 4:** Results of the binary logistic regression analysis with simultaneous entry.

Variable						95% Confidence Interval	
	Beta	SE	Wald	p	odds ratio	Lower Bound	Upper Bound
Attitudes	−0.301	0.271	1.238	0.266	0.740	0.435	1.258
Perceived Usefulness	0.490	0.274	3.193	0.074	1.632	0.954	2.791
Perceived Ease of Use	0.150	0.143	1.101	0.294	1.162	0.878	1.537
Compatibility	0.096	0.166	0.339	0.560	1.101	0.796	1.524
Social Norms	−0.225	0.204	1.218	0.270	0.798	0.535	1.191
Client Influence	−0.161	0.169	0.903	0.342	0.851	0.611	1.187
Peer Influence	−0.001	0.186	0.000	0.997	0.999	0.694	1.439
Superior Influence	0.378	0.173	4.758	0.029^*^	1.459	1.039	2.048
Perceived Behavioral Control	0.977	0.175	31.254	<.001^*^	2.656	1.886	3.741
Self-Efficacy	0.037	0.156	0.056	0.813	1.038	0.764	1.409
Facilitating Conditions & Barriers	−0.244	0.304	0.642	0.423	0.784	0.432	1.423

Note.

* p < .05.

Following survey completion, participants were presented with an informational video regarding VR use in clinical practice. Afterwards, 278 (42.3% of the respondents) reported that they plan to use VR in their future clinical practice. Likewise, 330 (50.2% of the respondents) indicated that they intended to seek more continuing education regarding using VR in clinical practice.

## Discussion

The purpose of this study was to identify the acceptance, adoption, and perceived barriers of use of VR in rehabilitation by physical therapy professionals including both PTs and PTAs. To do this, licensed clinicians were surveyed using theory-driven questions regarding acceptance and usage of VR. Our results are discussed below in the context of previous findings and the associated theoretical framework.

A small percentage of respondents reported currently using VR in their clinical practice. On the ADOPT-VR2, 14.1% (n = 93) of licensed clinicians reported exploring VR in their clinical workplace. However, only 7.1% (n = 47) of licensed clinicians reported that they are currently using VR in their clinic. This is lower than previously reported, as 12% of Canadian clinicians and 31% of US clinicians reported using VR in their clinical practice [14,15]. This difference in reported VR usage by clinicians in the US could be due to sample differences. First, this previous research surveyed both PTs and OTs, and the total sample for the survey previously conducted in the US was 552 respondents versus the total sample of 658 in the current survey. The authors also cited that their sampling strategy relied heavily on personal contacts and social media, which could have possibly skewed the respondent pool toward those with more experience using VR. The anonymous nature of our survey along with our sampling strategy does not afford us the knowledge of the number of total potential participants reached. Moreover, as a limitation, the survey did not have many safeguards against the potential of multiple responses. However, the largest number of direct email contacts (51,370) came via email lists received by the licensing boards of Ohio (20,396), North Carolina (13,984), Arizona (7,896), Louisiana (5,054), Maine (2,993), and Wyoming (1,047). Our method may have allowed for recruitment of a sample with less bias toward current VR use, resulting in lower reported percentages of experience with VR and current VR usage than previous reported [14,15].

Lower cost, commercially available VR was more commonly used compared to medical-grade products. This is consistent with previous research in which respondents reported using primarily the Wii Fit or Xbox Kinect, as well as systematic reviews of VR research which also demonstrate commercial grade products are more commonly used by to perform VR interventions [2,3,14,15]. The low-cost, low profile, and easy setup of commercial product like Nintendo Wii are qualities often cited as favorable factors over medical-grade VR systems [9,16]. However, a drawback of commercial products is that the programs associated with the system are primarily created for entertainment and recreational purposes, which requires clinicians to adapt the use of these programs to fit the desired outcome of their physical rehabilitation intervention [4,24]. Future research should aim to leverage the accessibility of commercially available VR products in their research to create evidence-based guidelines for the use of low cost, commercially available VR systems with adaptations for rehabilitation.

The constructs of Compatibility, Attitudes, Self-Efficacy, Perceived Ease of Use, and Client Influence were found to significantly contribute to the prediction of intention to adopt VR in clinical settings (i.e., Behavioral Intention). Compatibility is the extent to which the clinician feels they can incorporate VR into their session, whereas Attitudes refers to their feelings toward using VR in clinical practice. Self-Efficacy is their confidence in using VR in the clinic, while Perceived Ease of Use is the extent to which the clinician feels they can operate the VR system with minimal effort. Lastly, Client Influence refers to the role a client has on persuading the physical therapy professional to use VR in their session. The partial correlations of these significant predictors were all positive, sans Perceived Ease of Use. For example, the first question in the Compatibility construct asks, “Using VR fits with the way I work” and the first question in the Attitudes construct asks, “Using VR in treatment sessions with my patients is a good idea”. Participants who answered closer to “strongly agree” to these questions also indicated stronger intention to include VR in clinical practice in the Behavioral Intention questions (i.e., a positive correlation). The strength of the correlations also provides insight into which of the significant constructs played a bigger factor in the intention to include VR in clinical practice, with Compatibility (partial r = .294) having the strongest correlation, followed by Self-Efficacy (partial r = .115), Attitudes (partial r = .101), Perceived Ease of Use (partial r =  -.096), and Client Influence (partial r = .086). Collectively, these data indicate that physical therapy professionals’ intention to adopt VR in clinical practice is more strongly connected to internal factors such as their values (Compatibility, Attitudes) and ability to use the VR equipment (Self-Efficacy, Perceived Ease of Use), rather than external constructs (e.g., Social Norms, Peer Influence, Superior Influence). These observations support the findings of Alrashidi et al. [21] and Cho et al. [22], both of whom observed Compatibility and Attitudes as significant predictors of Behavioral Intention to use VR in clinical practice using a similar statistical model. Interventions that target these factors could be useful in increasing VR use as an intervention in clinical settings. An intervention to address these constructs may look like an in-service either specifically for clinical supervisors, or for both supervisors and staff, discussing support for VR interventions, types of VR, and actionable ways to initiate VR usage in their clinical setting.

Following the demographic and ADOPT-VR2 portions of the survey, participants were shown an informational video regarding VR use in clinical practice. Although only 7.1% of the respondents reported that they are currently using VR in clinical practice, after watching the short informational video on ways that VR could be incorporated into the clinic, 42.3% of the respondents indicated they plan to use VR in the future. This remarkable increase in VR interest indicates that short educational sessions may be key to helping support their infusion of VR into clinical practice. Likewise, 330 of the 658 respondents (50.2%) reported that they intend to learn more about VR after watching the informational video, supporting the assertation that there is a desire by a critical mass of physical therapy professionals to consider using VR in clinical practice. This speaks to the importance of the behavioral factors found in our secondary analysis to be significant predictors of positive intention and adoption of VR interventions clinically, especially the factor of Perceived Behavioral Control. Perceived Behavioral Control relates to factors such as having the knowledge on how to incorporate VR, having access to resources related to using VR, being familiar with the current evidence on the efficacy of VR, and knowing what VR games are available for using clinically.

These factors could be addressed through additional education related to VR and clinical VR interventions. It should be noted that an educational approach to enhance knowledge translation in support of the implementation of VR in clinical practice has shown positive benefits [25]. In this five-month longitudinal study, therapists were provided support via one-on-one training, small group learning, and VR game descriptions to enhance their VR knowledge in clinical translation. While this was a necessary first step, the small sample size (n = 11) limits generalizability. Additional research using longitudinal designs to track changes in intention and actual VR usage over time to assess the long-term impact of interventions aimed at influencing VR acceptance and adoption would be useful for understanding the future potential of VR use in clinical practice.

While our study design provided data to identify the acceptance, adoption, and perceived barriers of use of VR in rehabilitation by physical therapy professionals, there are some key factors that were not addressed with our survey. For example, if VR is going to be adopted more widely in clinical practice, the utility of VR relative to traditional therapy approaches will need to more clearly described. It has been shown that VR can lead to equal to or better motor behavior outcomes in the short-term [2–7,26], but the extent to which engaging in VR-enhanced physical therapy helps with long-term motor skill development and/or retention after therapy has not yet been examined. Further, the motivational benefits relative to engagement in therapy that infuses VR needs to be more fully described. Examining these issues in study designs with a head-to-head comparison between traditional therapy approaches and VR-enhanced approaches will help define the extent to which the cost of VR (financial, time, effort) is outweighed by the benefits (e.g., physical, motivational).

It should be noted that some limitations to this study exist. First, due to lack of control over the total reach of recruitment for this survey, which was conducted via email and other digital outlets, the sample may not be fully representative of the larger population of PT and PTA clinicians. Additionally, the survey had minimal safeguards against multiple entries. Further, an operational definition of “virtual reality” was not provided prior to taking the survey, leaving open the possibility of different interpretations of that term and its implementation in clinical practice. Lastly, while participants were shown an informational video on VR use in clinical practice, they were not asked the same questions pre- and post-video and therefore it’s difficulty to conclusively determine if the video had any impact on clinicians’ intentions toward adopting VR in their clinical practice.

To our knowledge, this survey is one of only a few surveys of this nature and scale investigating the use of VR by rehabilitation professionals, and the first to incorporate PTAs. The views of licensed clinicians provide important insight into current and future usage of VR in clinical practice. Future research using interventions that specifically address the most influential factors related to VR usage in clinical practice would be useful to further address the gap between evidence and clinical practice. This study found that Superior Influence and Perceived Behavioral Control were the most influential factors in VR usage among participants that reported actually using VR in their clinical practice. Creating interventions targeting these factors may be key in improving the translation of behavioral intention into practice.

## Conclusions

Most PT and PTA respondents reported they currently do not use VR in clinical practice, but a critical mass are open to exploring its use in the future. Factors influencing the adoption of VR in clinical practice were identified, so future research should develop interventions that focus on these factors to examine the extent to which VR adoption in clinical practice is altered in the short and long-term.

## Supporting information

S1 Link Link to the informational VR video provided to participants.
(DOCX)

S2 Table Clinician survey responses.
(XLSX)
