# Supplementary material for: Clinician perspectives on virtual reality use in physical therapy practice in the United States
Source: PLoS One. 2025 Apr 2;20(4):e0320215. doi: 10.1371/journal.pone.0320215 (PMC11964245; doi:10.1371/journal.pone.0320215)
Supplement: S1 Link — (DOCX) [file pone.0320215.s001.docx]

https://www.youtube.com/watch?v=Px5krM_eUqY
